# A Patient with Fibroepithelial Polyp of the Ureter—A Rare Condition Mimicking Malignancy: A Case Report

**DOI:** 10.1155/2012/901693

**Published:** 2012-12-27

**Authors:** Wolfgang Brummeisl, Hans-Martin Fritsche, Elisabeth Huber, Wolf F. Wieland, Roman Ganzer

**Affiliations:** ^1^Department of Urology, University of Regensburg, 93053 Regensburg, Germany; ^2^Caritas St. Josef Medical Center, University of Regensburg, 93053 Regensburg, Germany; ^3^Department of Pathology, University of Regensburg, 93053 Regensburg, Germany

## Abstract

A 61-year-old man presented with hematuria and intermittent right pelvic pain. Intravenous urography showed a tubular filling defect and ureteroscopy a tumor in the right mid ureter. Urine cytology and tumor biopsy showed nonmalignant results. Open surgery was performed, and an intraoperative frozen section revealed a fibroepithelial polyp of the right mid ureter. A fibroepithelial polyp is a rare benign lesion that can occur in childhood but is an important differential diagnosis of an upper urinary tract urothelial cell carcinoma in adults.

## 1. Background

The upper urinary tract urothelial cell carcinomas (UUT-UCCs) account for only 5–7% of UCCs. Of these only 25% are located in the ureter. In western countries, the incidence is 1-2 cases per 100,000 per year [1]. Typical symptoms are gross hematuria (80%) and flank pain (30%) and often caused by clots passing down the ureter. Males are affected three times as common as women. The incidence increases with age and smoking confers a two-fold risk [1]. Diagnosis is usually made combining with urine cytology, intravenous urography (IVU), or multidetector computed tomographic urography. Ultrasound is excellent for detecting renal parenchymal tumors, but not tumors of the renal pelvis or ureter. Further investigations are selective ureteric urine cytology, retrograde pyeloureterography, or flexible uretero-renoscopy [1]. The gold standard treatment for invasive UUT-UCCs, regardless of the location of the tumor in the UUT, is radical nephroureterectomy (RNU) with excision of a bladder cuff. We describe the case of a patient with symptoms and findings mimicking features of an UUT-UCC.

## 2. Case Presentation

**Figure 1:**
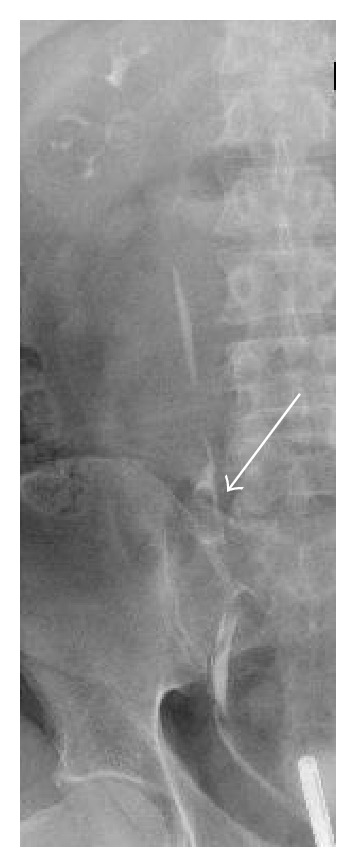
Retrograde pyeloureterography revealing right mid-ureteral filling defect.

**Figure 2:**
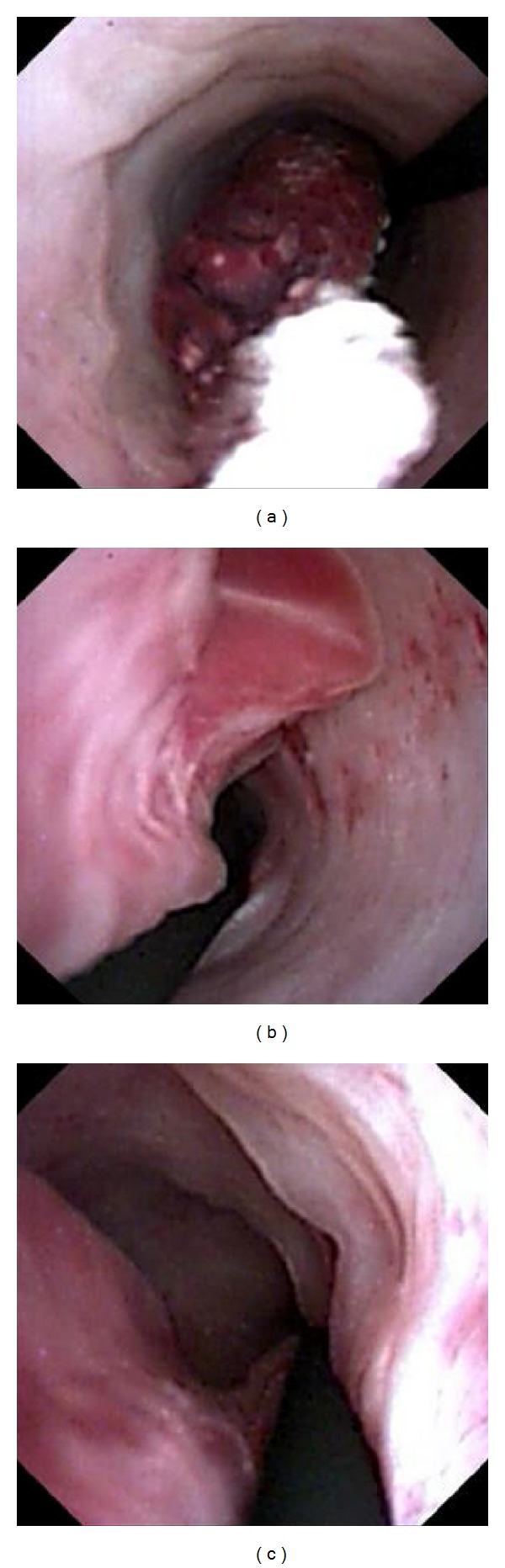
Ureteropyelography presenting a 5 cm long tumor in the right mid ureter (distal-mid-proximal part); inlaying guide wire.

**Figure 3:**
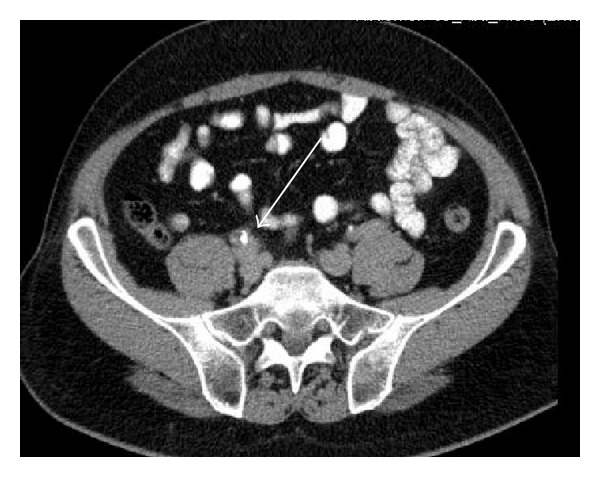
Thin-layer computed tomography demonstrating a small thickening of the right mid ureter.

**Figure 4:**
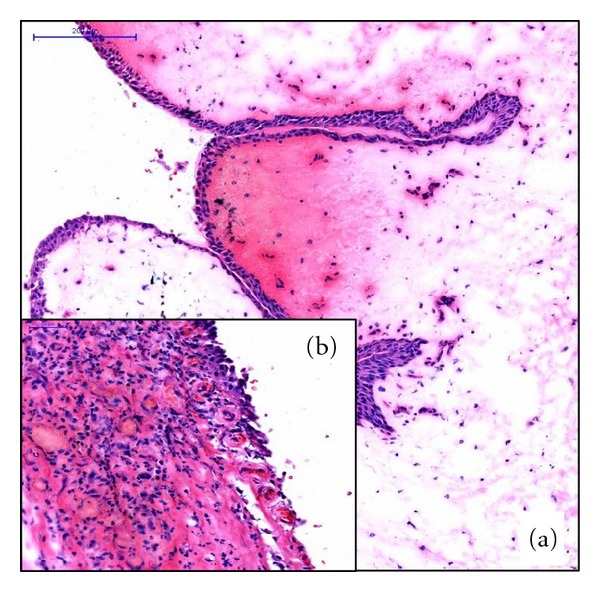
HE staining showing a fibroepithelial polyp with covering urothel (a), some erosion, and inflammation (b), but no atypia or malignancy.

A 61-year-old man presented to our department with symptoms of gross hematuria and intermittent right pelvic pain. He had a past medical history of arterial hypertension, psoriasis, an annealed lung Tbc, and a smoking history of 60 pack years. Urine analysis showed microscopic hematuria. Biochemical data were within normal limits. Ultrasound and cystoscopy were normal. Intravenous urography (IVU) demonstrated a tubular filling defect with partial obstruction of the right mid ureter. Retrograde ureteropyelography confirmed a 5 cm long tubular mass in the mid ureter proximal to the iliac vessels (Figure 1). Further investigation was done by flexible ureteroscopy showing a pediculated tumor of the ureter with a vulnerable surface (Figure 2). Urine cytology and biopsy of the tumor and the renal pelvis were taken. A pigtail catheter was inserted. Results of urine cytology and the biopsy were negative for malignancy. A staging CT scan was performed which showed a nonspecific slight dilatation of the right renal pelvis and a small thickening of the right ureter without evidence of metastasis or lymphadenopathy (Figure 3). Open exploration of the right ureter was performed by a Gibson incision. After palpating the mass above the level of the iliac vessels, an ureterotomy was made. The tumor presented with a small thin basis and a length of 5 cm. Intraoperative frozen section revealed a benign fibroepithelial polyp (UFP) without evidence of malignancy (Figure 4). Segment resection of the ureter was performed followed by a tension-free spatulated end-to-end anastomosis. The patient was discharged from hospital three days postoperatively with a foley catheter in place, which was removed at day 10. The pigtail catheter was removed 5 weeks later. 

## 3. Discussion

A fibroepithelial polyp of the ureter is a rare cause of hematuria and imaging findings that make it difficult to differentiate from UUT-UCCs, which are usually treated by RNU. A differential diagnosis between UUT-UCCs and UFP cannot be made with imaging tests only. Urine cytology and endoscopic biopsy alone might be insufficient to confirm the diagnosis as shown in our case report. 

A UFP is a rare benign mesodermal tumor with approximately 200 cases documented in the literature. In recent years, most cases are reported in Asian children, but older reviews show that it can occure in every age and not only in Asia [2, 3]. 

Histologically, these polyps are composed of stroma derived from the mesoderm and covered by a layer of normal transitional epithelial cells [4]. Most UFP are long slender projections with a smooth surface arising from a small base. These polyps are thought to be either congenital slow-growing lesions or lesions that develop as a result of chronic urothelial irritants, such as infection, inflammation, or obstruction. Other benign lesions of the upper urinary tract include endometriomas, fibromas, leiomyomas, granulomas, neurofibromas, hemangiomas, and lymphangiomas [5, 6]. 

UFP can occur in every age, but commonly they present in the third to fourth decades of adults. Males are 1.5 times more affected with it than females. Most UFP occur in the left ureter with a predilection for the proximal segment. Mean fibroepithelial polyp diameter is reported with less than 5 cm; however, larger polyps that even can protrude into the bladder have been reported [7, 8]. Ureteral polyps usually appear as solitary tumors; however, rare cases of multiple and bilateral appearance have been reported [2].

Most common symptoms of patients with UFP are gross hematuria and intermittent right pelvic pain. Some cases are presented with hydronephrosis. IVU or retrograde pyelography typically demonstrates a tubular filling defect. This finding combined with negative cytology and biopsy should suspect a UFP. There is no evidenced guideline for the treatment of a UFP; however, priority should be the complete excision of the tumor with minimal risk of morbidity and maximal preservation of renal function. 

Despite no preoperative evidence of malignancy, many case reports of UFP include management by open or laparoscopic surgery. Recent publications refer endoscopic percutaneous or ureteroscopic approach excisions or laser coagulation of the polyp [2, 9]. Endoscopic treatment means no dermal scars, less hospital stays, and reduced burdens for the patient. But like in our case, endoscopic surgery can be limited. The poly can constrict the ureter so that it is impossible to pass or access the base of the polyp. View also can be limited by little working space or bloody urine, so that open or laparoscopic approach with segmental resection of the ureter is necessary [2]. 

## 4. Conclusion

A fibroepithelial polyp of the ureter is a rare benign tumor mimicking typical findings of UUT-UCCs. It has to be included in the differential diagnosis of UUT-UCCs, especially when urine cytology and biopsy are negative. In this condition, nephroureterectomy would be malpractice.
